# Carboxyhaemoglobin levels and their determinants in older British men

**DOI:** 10.1186/1471-2458-6-189

**Published:** 2006-07-18

**Authors:** Peter Whincup, Olia Papacosta, Lucy Lennon, Andrew Haines

**Affiliations:** 1 Division of Community Health Sciences, St George's, University of London, London SW17 0RE, UK; 2 Department of Primary Care & Population Sciences, UCL, Hampstead Campus, London NW3 2PF, UK; 3 Director's Office, London School of Hygiene and Tropical Medicine, Keppel Street, London WC1E 7HT, UK

## Abstract

**Background:**

Although there has been concern about the levels of carbon monoxide exposure, particularly among older people, little is known about COHb levels and their determinants in the general population. We examined these issues in a study of older British men.

**Methods:**

Cross-sectional study of 4252 men aged 60–79 years selected from one socially representative general practice in each of 24 British towns and who attended for examination between 1998 and 2000. Blood samples were measured for COHb and information on social, household and individual factors assessed by questionnaire. Analyses were based on 3603 men measured in or close to (< 10 miles) their place of residence.

**Results:**

The COHb distribution was positively skewed. Geometric mean COHb level was 0.46% and the median 0.50%; 9.2% of men had a COHb level of 2.5% or more and 0.1% of subjects had a level of 7.5% or more. Factors which were independently related to mean COHb level included season (highest in autumn and winter), region (highest in Northern England), gas cooking (slight increase) and central heating (slight decrease) and active smoking, the strongest determinant. Mean COHb levels were more than ten times greater in men smoking more than 20 cigarettes a day (3.29%) compared with non-smokers (0.32%); almost all subjects with COHb levels of 2.5% and above were smokers (93%). Pipe and cigar smoking was associated with more modest increases in COHb level. Passive cigarette smoking exposure had no independent association with COHb after adjustment for other factors. Active smoking accounted for 41% of variance in COHb level and all factors together for 47%.

**Conclusion:**

An appreciable proportion of men have COHb levels of 2.5% or more at which symptomatic effects may occur, though very high levels are uncommon. The results confirm that smoking (particularly cigarette smoking) is the dominant influence on COHb levels.

## Background

Carbon monoxide (CO) is produced by the incomplete combustion of carbon-containing material; important sources include tobacco, biomass fuels (e.g. wood) and fossil fuels (e.g. natural gas, coal, petrol, diesel). CO displaces oxygen from haemoglobin in red cells to produce carboxyhaemoglobin (COHb), which acts as a sensitive and specific marker of atmospheric carbon monoxide exposure from both indoor and outdoor sources [1].

Although the toxic effects of acute high concentrations of CO have been recognized for many years, there has been increasing concern that prolonged exposure to low levels of CO may have adverse health effects, particularly cardiovascular and neurophysiological[2]. The adverse cardiovascular consequences reported at COHb levels of 2–5% include a diminution in exercise tolerance, both in healthy individuals[3] and in those with ischaemic heart disease[4,5]. Chronic CO exposure in ambient air pollution may also increase the risk of developing heart failure[6,7]. It has also been suggested that increased levels of CO might contribute to the development of coronary heart disease[8,9], possibly though effects on platelet and endothelial functioning[10], though this remains speculative[11]. Cognitive function may also be impaired at COHb levels of 5% or so [12-14]. Case reports have suggested that long-term neurological effects may occur[15], but this has not been examined in long-term epidemiological studies[1].

Although there is an extensive literature on CO poisoning[16], information on the extent and the main determinants of CO exposure in the British population is limited. Earlier personal exposure studies have suggested that indoor sources including cigarette smoke and gas cookers make important contributions to CO exposure [17] and to COHb levels[18], while the contribution of outdoor sources is modest[16]. However, there is little information about the levels of COHb prevalent in the British population and its determinants, which is of particular concern because of the widespread use of gas heating appliances in Britain [1]. Information on older subjects is particularly important because they spend more time at home than younger age-groups and are therefore at particularly high potential risk. We report on a population-based study of COHb levels carried out in men aged 60–79 years during the 20 year follow-up examination of the British Regional Heart Study cohort, which provided an opportunity to examine seasonal, regional, social, household and individual determinants of COHb levels.

## Methods

The British Regional Heart Study is a prospective study of cardiovascular disease among middle-aged and older men. In 1978–80, a stratified random sample of 24 medium-sized towns (50,000–125,000 population not part of major conurbations) in England, Wales and Scotland was selected, ensuring representation of all major regions [18]. A random sample of 400 men aged 40–59 years was drawn from one socially representative group General Practice in each town. In all, 7735 men (78% response rate) were recruited into the study and followed up both through the NHS Central Register and through their General Practitioner since their initial assessment (tracing rate 99%). Between 1998 and 2000, all surviving men, then aged 60–79 years, were invited for a 20 year follow-up examination, carried out in a local health centre or other similar accommodation. The study obtained ethical approval by the London Multi Research Ethics Committee (ref MREC/02/2/91). Ethical approval was also obtained from all the relevant twenty two local research ethics committees and written informed consent was sought from all participants. Subjects were measured in their original town of examination, unless (particularly in the case of migrants) they preferred to be measured in another study town nearer to their current place of residence. Towns were visited in rotation between February 1998 and February 2000. Seasons of measurement were defined as winter (Dec-Feb), spring (Mar-May), summer (Jun-Aug) and autumn (Sep-Nov). All participants completed a questionnaire providing information on their medical history, smoking habits, current employment status, most recent occupation, housing tenure and on their domestic heating and cooking arrangements – providing separate details of the fuels used for heating and cooking. Subjects were asked to recall doctor diagnoses of cardiovascular disease (including myocardial infarction, angina, stroke, peripheral arterial disease). Smoking consumption was classified into 8 groups including never, ex, current pipe or cigar and cigarette smokers. Subjects smoking both pipe/cigar and cigarettes were classified as cigarette smokers. 'Light' pipe and cigar smokers were those smoking ≤ 10 cigars or 30 grams of pipe tobacco per week; those smoking more were classified as 'heavy'. Subjects who reported exposure to other peoples cigarette smoke, for at least 1 hour, at or outside their home were classified as passive smokers. Social class was defined from longest-held occupation using the Registrar General's 1980 coding manual into 3 non-manual and 3 manual categories.

A team of three research nurses made physical measurements and collected a fasting blood sample. A whole blood sample collected in fluoride oxalate after a six hour fast was transported overnight to a single central laboratory for analysis within 36 hours of collection. COHb was measured using a co-oximeter (AVL Medical Instruments, Ltd) which was calibrated with each batch of samples and was registered in an external quality assurance programme. The lower limit of detection was 0.2% and the coefficient of variation at a COHb concentration of 2.0% was 0.05. There were 257 (7.1%)subjects with undetectable COHb levels. The distribution of carboxyhaemoglobin values was markedly skewed. Log transformation (with 0 values set at 0.05%) reduced skewness considerably. Geometric means and 95% confidence intervals have been used throughout.).

All statistical analyses were carried out using the SAS programme (version 6.12). All adjusted means presented in Tables 1, 2, 3, 4 were computed using the LSMEANS option within PROC GLM; all explanatory variables were fitted as class variables with the appropriate number of levels. The p values presented refer to the results of statistical tests for heterogeneity in COHb levels between the explanatory variable categories.

## Results

**Figure 1:**
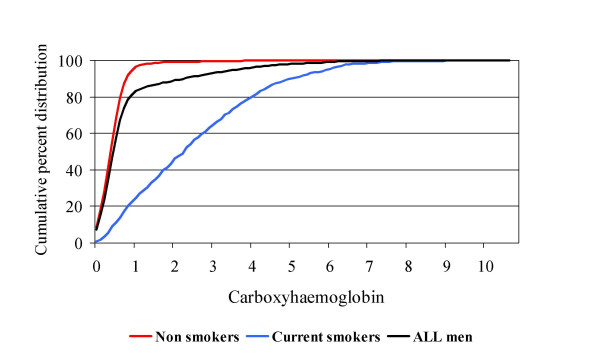
Distribution of COHb levels in older men. Values for all men, current smokers and non-smokers are shown separately.

Of 5565 surviving subjects, 4252 (76%) attended for examination; 4025 (72%) had COHb measurements made. Because it was possible that men who had travelled appreciable distances for examination would have COHb levels that did not reflect their habitual exposure, the analyses are based on 3603 subjects who lived in or within 10 miles of the town in which they were examined. The distribution of COHb levels in the whole study population was skewed to the right; skewing was concentrated among smokers (Figure 1). Among the whole study population the geometric mean COHb concentration was 0.46% and the median concentration 0.50% (interquartile range 0.30 to 0.80%); geometric mean and median concentrations were 0.33 and 0.4 (IQR 0.2 to 0.6) in current non-smokers, 1.83 and 2.3 (IQR 1.1 to 3.7) among current smokers. Among the whole study population, COHb levels of 2.5% or more were observed in 330 men (9.2%), levels of 5% or more in 72 men (2%) and levels of 7.5% or more in 5 men (0.1%). Mean COHb level fell slightly with increasing age, from 0.47% in the 60–64 year age-group to 0.43% in the 75–79 year age-group (test for trend; p = 0.06). Overall, mean COHb levels fell slightly between morning and afternoon. However, diurnal variation differed between non-smokers (who showed a proportional fall between morning and afternoon of 25%, 95% CI 19.5 to 29.2%) and smokers, who showed a proportional rise of 6.0%, 95% CI -7.0 to 21.6%); there was strong evidence of a smoking*time of day interaction (p = 0.001). There was marked seasonal variation in mean COHb levels, which were higher in the autumn [September-November] and winter [December-February] quarters (0.53 and 0.54% respectively) than in spring [March-May] and summer [June-August] quarters (0.38, 0.39 % respectively); a test for seasonal differences was highly statistically significant (p < 0.0001).

The relations between region of residence, social class, employment status and housing tenure and COHb levels (standardized for age, time of day and season of measurement) are shown in Table 1 (Model 1). Geometric mean COHb levels were lowest in Southern England and highest in Northern England. There was a strong social class gradient, with lower COHb levels in non-manual occupations. Subjects describing themselves as unemployed had markedly higher COHb levels than those who were employed or retired, who had similar levels. Housing tenure was strongly related to COHb levels, with the lowest levels observed in owner occupiers and markedly higher levels among those living in rented accommodation.'

The relations between active and passive smoking, domestic factors (use of gas for cooking or heating, presence or absence of central heating or double glazing) and COHb levels are shown in Table 2 (Model 1). Non-smokers and ex-smokers had similar COHb levels. Compared with non-smokers, current pipe and cigar smokers showed a graded rise in COHb levels, with a difference of about fivefold between the heaviest smokers and non-smokers. Current cigarette smokers showed a stronger graded rise, with a difference of about tenfold between the heaviest smokers and non-smokers. Exposure to the tobacco smoke of others was associated with a more modest proportional increase in COHb level of slightly more than a half. The use of gas for cooking was associated with a small proportional increase in COHb level; the use of gas for heating showed no relationship with COHb level. Subjects with central heating had lower mean COHb levels than those without; the presence or absence of double glazing was not related to COHb level. Among those without central heating, most were using gas heating alone (48%), electricity alone (16%) or both (27%); few (8%) used neither. Among these, electricity users had slightly lower COHb levels (0.56%) compared with the other groups which were all similar (0.68%).

Many of the factors related to COHb level in univariate analyses were inter-related. The prevalence of current smoking varied markedly by region, social class, employment status and housing tenure, and was higher among subjects who did not use gas for cooking or have central heating (Tables 1 and 2). The independent relationships between each factor and COHb level, adjusted for all other factors in these tables which had statistically significant univariate associations with COHb, are presented in the right hand (Model 2) columns of Tables 1 and 2. The associations between employment status, housing tenure, social class, passive smoking exposure and COHb were markedly reduced or abolished by adding adjustment for other statistically significant determinants of COHb level. The associations between gas cooking, central heating and COHb were reduced after adjustment but remained statistically significant. The relations between region, active smoking and COHb remained strong and highly statistically significant after adjustment. When the analyses presented in Tables 1 and 2 were repeated among current non-smokers, the findings were very similar (Table 3). Region, social class, gas cooking and central heating showed associations with COHb (Model 1) which persisted after adjustment for other determinants of COHb level (Model 2). The associations between employment status, housing tenure, gas heating, double glazing, passive smoking and COHb (Model 1) did not remain statistically significant after adjustment for other statistically significant determinants of COHb level (Model 2).

Active smoking alone accounted for 41% of variance in COHb levels in the study population; all the factors examined together accounted for 47% of variance in COHb level. The prevalence of active smoking (cigarette, pipe or cigar) rose steeply at increasing COHb thresholds. At levels of >0.5%, >1.0%, >2.5%, >5.0% and >6.0% the prevalences of active smoking were respectively 39, 83, 93, 97 and 100% respectively.

The relationships between COHb levels and prevalent vascular disease (based on recall) are presented in Table 4. There was no strong association between myocardial infarction and COHb level. Men with angina, stroke and peripheral vascular disease all had slightly higher mean COHb levels than men without, though only for peripheral vascular disease was the difference statistically significant. After adjustment for cigarette smoking prevalence (lower among men with myocardial infarction and angina, higher among men with stroke and peripheral disease) and for the other factors related to COHb level (Tables 1 and 2), difference in COHb levels were markedly reduced, except for men with angina in whom the differences were of marginal statistical significance. Appreciable proportions of men with these conditions had COHb levels of 2.5% or more (12.4%, 11.1%, 7.5%, 7.3% respectively for peripheral arterial disease, stroke, angina and myocardial infarction).

## Discussion

To the best of our knowledge, this is the first published report describing the levels of COHb in a population-based sample of older British adults. Average COHb levels in this study population (0.3% in non-smokers and 1.8% in smokers) were appreciably lower than those observed in studies among slightly younger adult populations in Scotland in the mid-1970s (approximately 1.6% in non-smokers and 5% in smokers) and in the United States in the late 1970s (approximately 0.8% in non-smokers and 4% in smokers) [19,20]. Although changes in COHb measurement between these surveys cannot be excluded, it is likely that the differences mainly reflect reductions in CO exposures influencing COHb levels, particularly outdoor exposure, which has fallen in the UK during the last 20 years[21].

However, despite the lower overall COHb levels, an appreciable proportion of subjects (almost 10%) had COHb levels of 2.5% or more, though the prevalences of markedly raised COHb levels, above 5.0% and 7.5%, were very small. Smoking (particularly cigarette smoking) is much the strongest determinant of high COHb levels, as in earlier reports [16,20]. The use of gas for cooking is associated with a modest increase in COHb level and the use of central heating with a modest decrease in individual levels. Although measurements were not made at home, the main analyses were restricted to subjects who were likely to have travelled directly from home to the measurement site. The assessments of COHb in these subjects should therefore provide a reasonable estimate of their ambient levels. Although the response rate in this survey of older men was relatively high, it is likely that the overall COHb values represent a slight underestimate, since non-responders are more likely to be from Northern England and Scotland and to be cigarette smokers when compared with responders [22]. It is likely that mean COHb levels (and the prevalence of high values) would be somewhat lower among women, in whom the prevalence and intensity of smoking would be expected to be lower; this is supported by the findings of a Scottish study [19].

Smoking, particularly cigarette smoking, was the strongest determinant of COHb levels. There was a strong dose-response relationship between number of cigarettes smoked and COHb level up to 20 cigarettes/day, with a plateau above this level. Although this could reflect inaccuracy in smoking reporting, the finding is consistent with the findings of an earlier study of British men measured in 1975–1982 – suggesting that at high cigarette consumption, inhalation per cigarette smoked decreases [23]. In the present study, the plateau occurred at around COHb levels of 3%, compared with 6% in the earlier study. This suggests that reported cigarette smoke intake does not equate directly with biological exposure and suggests that overall cigarette smoke exposure, particularly at high cigarette consumption, may have declined over time. The findings could also be consistent with the results of a recent study suggesting that among smokers the level of COHb may provide independent prediction of cardiovascular risk, even after taking amount smoked into account[19]. The absence of any consistent association between environmental tobacco smoke exposure and COHb is consistent with earlier reports[24].

Among other determinants of COHb levels in individuals, the associations with use of gas cooking and central heating were observed both among the whole study population and also among non-smokers, supporting the validity of the findings. The association between use of gas cooking and higher mean COHb levels is consistent with the findings of earlier reports showing that the use of gas cooking was associated with marked increases in environmental CO concentrations and with higher mean levels of COHb[16]. However, the influence of gas cooking on population levels of COHb is modest. The association between central heating and lower COHb level (with no apparent relation between type of heating and COHb) probably reflects the overriding importance of the quality of venting for heating appliances. Central heating appliances, with their purpose-built ventilation flues, appear to be protective. Among the relatively small number of participants without central heating, levels of COHb appeared somewhat lower in those using electrically powered heating appliances than in those using combustion appliances of any kind. The small number of participants without central heating makes it difficult to discriminate between the effects of different fuels in this setting. The presence of double glazing did not appear to affect COHb levels – a finding consistent with earlier reports of domestic determinants of CO levels[25]. However, our data on double-glazing are crude; it remains possible that CO levels are higher in homes with particularly high-quality glazing.

Regional differences in COHb have previously been reported, particularly in relation to degree of urbanization[20]. In the present study, in which all subjects lived in medium sized population centres, average COHb levels were appreciably lower in Southern England than in other regions. Although there are appreciable differences in the prevalence of cigarette smoking between regions, other determinants (particularly central heating and gas cooking) did not show strong corresponding regional patterns (data not shown); adjustment for these factors did not appreciably reduce regional variation in COHb levels, either among all subjects or among non-smokers. Variations in outdoor CO exposure may well be important in accounting for the regional differences in COHb levels. Further analyses based on information on variations in CO emissions and exposures, which occur particularly in relation to transport facilities [21], would allow this issue to be examined further.

The factors examined here account for approximately half of the variation in COHb observed. Some of the unexplained variance is likely to be explained by imprecision in the assessment of exposure. This is likely to apply particularly to active smoking exposure, which influences COHb to an extent strongly determined by the degree of inhalation [23] and to gas cooking, to which the amount and intensity of exposure in likely to vary considerably. It is also likely that outdoor exposure accounts for a proportion of unexplained variance. Outdoor exposure is largely from combustion of fossil fuels used by road traffic. This may occur by direct outdoor exposure and by the indirect effects of outdoor CO levels on indoor levels. Though most studies have suggested that indoor CO levels are higher than those outdoors[16], this has not been the case in all studies[26], suggesting that outdoor levels may influence indoor CO exposure. No information on outdoor traffic-related exposures are available for this study population.

Although earlier studies have suggested that CO exposure might increase the risk of developing cardiovascular disease, the information on COHb levels and prevalent disease is difficult to interpret. The acquisition of a cardiovascular diagnosis may have reduced CO exposure, especially by inducing changes in smoking habit. This is particularly likely to have occurred among subjects with MI and angina, where the prevalence of smoking is lower among cases than non-cases. Although subjects with MI, angina and peripheral arterial disease tended to have slightly higher COHb levels than those who did not have these diagnoses, the differences were not marked and they do not constitute strong evidence for a specific causal association between COHb and the development of vascular disease. However, appreciable proportions of subjects with vascular disease had levels of COHb above 2.5%, suggesting that suggest that there may be substantial opportunities for the improvement of exercise tolerance among these subjects by reducing COHb levels. The reduction in smoking levels which would be the principal means of bringing about such reductions would also have important direct benefits for the prevention of cardiovascular disease[27].

## Conclusion

The results confirm that smoking (particularly cigarette smoking) is the dominant influence on COHb levels. Markedly raised levels of COHb not associated with smoking appear to be uncommon (at COHb of 2.5% or above, 0.6% of the total population, at COHb of 5.0% or above, 0.06% of the total population). However, these estimates are not very precise, given the limited numbers of subjects affected at these very low prevalences. More detailed examination of these subjects suggested that they all had other non-tobacco exposures, particularly the use of gas cooking. However, it was not possible in this study to establish the contribution of badly functioning gas appliances in these individuals. Overall however these results suggest that the prevalence of high level exposure to carbon monoxide from non-tobacco sources is uncommon, even in this older and therefore high risk population.

## Abbreviations

Carbon monoxide (CO), Carboxyhaemoglobin (COHb)

## Competing interests

The author(s) declare that they have no competing interests.

## Authors' contributions

PW and AH raised funds for the study, which was planned by PW with assistance from LL, OP and AH. OP carried out the statistical analysis and PW wrote the paper, with the assistance of all other authors. All authors read and approved the final manuscript.

## Pre-publication history

The pre-publication history for this paper can be accessed here:



## Figures and Tables

**Table 1:** COHb levels in older men: relations to region, social class, employment status, housing tenure

			Model 1				Model 2			
		current smoking	COHb				COHb			

	N	%	Geometric mean	95%	CI	p	Geometric mean	95%	CI	p

Region of current residence						<0.0001				<0.0001
South	1155	16.5	0.35	0.33	0.38		0.37	0.35	0.39	
Midland+Wales	578	18.9	0.52	0.47	0.56		0.51	0.47	0.55	
North	1478	20.0	0.53	0.50	0.57		0.52	0.49	0.54	
Scotland	392	19.5	0.49	0.44	0.55		0.47	0.42	0.51	
										
Social Class						<0.0001				0.0344
I	274	9.9	0.37	0.32	0.42		0.46	0.42	0.51	
II	882	14.9	0.44	0.41	0.47		0.48	0.46	0.51	
IIIN	472	16.5	0.44	0.40	0.48		0.46	0.42	0.49	
IIIM	1197	20.6	0.47	0.44	0.50		0.44	0.42	0.49	
IV	475	25.3	0.56	0.51	0.62		0.48	0.44	0.51	
V	130	23.4	0.49	0.41	0.59		0.40	0.34	0.46	
										
Employment						0.0178				0.8007
Unemployed	77	29.0	0.64	0.50	0.83		0.46	0.37	0.56	
employed – (full or part time)	613	19.3	0.44	0.40	0.49		0.47	0.43	0.50	
Retired	2834	18.2	0.46	0.44	0.48		0.45	0.43	0.47	
										
Housing tenure						0.0001				0.6273
owner occupier	3041	16.3	0.43	0.42	0.45		0.45	0.44	0.47	
renting from local authority	342	33.4	0.68	0.60	0.76		0.47	0.43	0.52	
renting privately	85	35.3	0.70	0.55	0.88		0.50	0.42	0.60	
Other	33	24.2	0.50	0.35	0.73		0.47	0.35	0.64	

Social class: (non manual) I professional, II intermediate, IIIN skilled non-manual, (manual) IIIM skilled manual, IV semi skilled manual, V unskilled manual

Model 1: Includes the relevant factor with adjustment for age, time of day, season.

Model 2: Includes the relevant factor with adjustment for age, time of day, season, and other factors which were statistically significant in the univariate analyses

(region, social class, employment status, housing tenure, active smoking, passive smoking, gas cooking, central heating). An interaction term for active smoking*time of day is also included in the model (see text).

p values presented refer to the results of statistical tests of heterogeneity in COHb levels between the explanatory variable categories

**Table 2:** COHb levels in older men: relations to active/passive smoking, heating and cooking fuel, double glazing

			Model 1				Model 2			
		current smoking	COHb				COHb			

	N	%	Geometric mean	95%	CI	p	Geometric mean	95%	CI	p

Active smoking						<0.0001				<0.0001
Non	1080	-	0.32	0.30	0.33		0.32	0.30	0.33	
Ex	1825	-	0.34	0.33	0.36		0.35	0.34	0.36	
Pipe or cigar (light)	76	-	0.60	0.50	0.72		0.61	0.50	0.74	
Pipe or cigar (heavy)	99	-	1.50	1.28	1.77		1.53	1.29	1.81	
Cigarette smoking <5/d	102	-	1.36	1.16	1.59		1.39	1.17	1.64	
cigarette smoking 5–10/d	126	-	2.29	1.98	2.65		2.18	1.87	2.53	
cigarette smoking 10–20/d	184	-	3.22	2.86	3.63		3.07	2.70	3.48	
cigarette smoking >20/d	51	-	3.29	2.62	4.13		3.18	2.52	4.01	
										
Passive smoking						<0.0001				0.2306
No	2620	12.8	0.40	0.38	0.42		0.45	0.44	0.47	
Yes	983	34.4	0.66	0.62	0.70		0.47	0.45	0.50	
										
Gas cooking						<0.0001				<0.0001
No	1394	16.3	0.40	0.38	0.42		0.42	0.40	0.44	
Yes	2083	20.2	0.51	0.48	0.53		0.48	0.47	0.50	
										
Gas heating						0.7934				0.9968
No	455	21.3	0.46	0.42	0.51		0.46	0.42	0.50	
Yes	3074	18.2	0.46	0.44	0.47		0.46	0.44	0.47	
										
Central heating						<0.0001				0.0083
No	340	29.6	0.64	0.57	0.71		0.52	0.47	0.57	
Yes	3201	17.5	0.44	0.43	0.46		0.45	0.44	0.46	
										
Double glazing						0.7657				0.8951
Yes	1778	18.6	0.45	0.43	0.48		0.45	0.44	0.47	
No	1594	18.9	0.46	0.44	0.49		0.46	0.44	0.48	
in part	169	16.9	0.45	0.38	0.53		0.47	0.41	0.53	

Model 1: Includes the relevant factor with adjustment for age, time of day, season.

Model 2: Includes the relevant factor with adjustment for age, time of day, season, and other factors which were statistically significant in the univariate analyses

(region, social class, employment status, housing tenure, active smoking, passive smoking, gas cooking, central heating). An interaction term for active smoking*time of day is also included in the model (see text).

p values presented refer to the results of statistical tests of heterogeneity in COHb levels between the explanatory variable categories

**Table 3:** COHb levels in non smoking older men: Relations to region, social class, employment status, housing tenure, passive smoking, heating and cooking fuel, double

		Model 1				Model 2			
		COHb				COHb			

	N	Geometric mean	95%	CI	p	Geometric Mean	95%	CI	p

Region of current residence					<0.0001				<0.0001
South	955	0.26	0.24	0.27		0.26	0.25	0.28	
Midland + Wales	465	0.39	0.36	0.42		0.38	0.35	0.41	
North	1172	0.39	0.37	0.41		0.39	0.37	0.41	
Scotland	313	0.32	0.29	0.36		0.33	0.30	0.37	
									
Social Class					0.018				0.0011
I	245	0.32	0.29	0.35		0.34	0.31	0.38	
II	746	0.34	0.32	0.36		0.35	0.33	0.38	
IIIN	384	0.35	0.32	0.38		0.36	0.33	0.39	
IIIM	943	0.32	0.30	0.33		0.31	0.29	0.33	
IV	352	0.37	0.34	0.41		0.35	0.32	0.39	
V	98	0.30	0.26	0.36		0.27	0.23	0.32	
									
Employment					0.373				0.6649
Unemployed	54	0.38	0.30	0.48		0.37	0.29	0.47	
employed – (full or part time)	492	0.32	0.30	0.35		0.34	0.31	0.37	
Retired	2302	0.33	0.32	0.35		0.33	0.32	0.35	
									
Housing tenure					0.071				0.3643
owner occupier	25302	0.33	0.32	0.34		0.33	0.32	0.34	
renting from local authority	224	0.37	0.33	0.41		0.35	0.31	0.40	
renting privately	55	0.41	0.33	0.51		0.39	0.31	0.49	
Other	25	0.30	0.21	0.42		0.33	0.23	0.47	
									
Passive smoking					0.019				0.0896
No	2270	0.33	0.32	0.34		0.33	0.32	0.34	
Yes	635	0.36	0.33	0.38		0.35	0.33	0.38	
									
Gas cooking					<0.0001				<0.0001
No	1160	0.29	0.28	0.31		0.30	0.29	0.31	
Yes	1660	0.36	0.35	0.38		0.36	0.35	0.38	
									
Gas heating					0.268				0.4189
No	354	0.32	0.29	0.35		0.35	0.32	0.38	
Yes	2499	0.34	0.32	0.35		0.33	0.32	0.34	
									
Central heating					0.002				0.0059
No	238	0.39	0.35	0.44		0.39	0.35	0.43	
Yes	2622	0.33	0.32	0.34		0.33	0.32	0.34	
									
Double glazing					0.988				0.9038
Yes	1436	0.33	0.32	0.35		0.33	0.32	0.35	
No	1286	0.33	0.32	0.35		0.33	0.32	0.35	
in part	138	0.34	0.29	0.39		0.34	0.30	0.40	

For social class definitions see Table 1

Model 1: Includes the relevant factor with adjustment for age, time of day, season.

Model 2: Includes the relevant factor with adjustment for age, time of day, season, and other factors which were statistically significant in the univariate analyses (region, social class, passive smoking, gas cooking, central heating).

p values presented refer to the results of statistical tests of heterogeneity in COHb levels between the explanatory variable categories

**Table 4:** COHb levels in older men: relations to prevalent cardiovascular disease.

			Model 1				Model 2			
		current smoking	COHb				COHb			

	N	%	Geometric mean	95%	CI	p	Geometric mean	95%	CI	p

Recall of doctor diagnosis of										
										
Myocardial infarction						0.5927				0.1744
Yes	382	15.7	0.47	0.42	0.53		0.48	0.44	0.53	
No	3147	19.0	0.46	0.44	0.47		0.45	0.44	0.47	
										
Angina						0.1972				0.0492
Yes	522	15.4	0.48	0.44	0.53		0.49	0.45	0.53	
No	3007	19.2	0.45	0.44	0.47		0.45	0.44	0.47	
										
Stroke						0.0644				0.9806
Yes	199	21.3	0.52	0.45	0.61		0.46	0.40	0.52	
No	3330	18.5	0.45	0.44	0.47		0.46	0.44	0.47	
										
Peripheral arterial disease						0.0030				0.2144
Yes	162	23.1	0.59	0.50	0.69		0.50	0.43	0.57	
No	3367	18.4	0.45	0.44	0.47		0.45	0.44	0.47	

Model 1: Includes the relevant factor with adjustment for age, time of day, season.

Model 2: Includes the relevant factor with adjustment for age, time of day, season, and other factors which were statistically significant in the univariate analyses

(region, social class, employment status, housing tenure, active smoking, passive smoking, gas cooking, central heating). An interaction term for active smoking*time of day is also included in the model (see text).

p values presented refer to the results of statistical tests of heterogeneity in COHb levels between the explanatory variable categories
